# High efficacy of brigatinib for brain metastases in ALK fusion gene‐positive non‐small cell lung cancer: A case series

**DOI:** 10.1111/1759-7714.15207

**Published:** 2023-12-30

**Authors:** Kei Morikawa, Yu Numata, Yusuke Shinozaki, Shotaro Kaneko, Aya Matsushima, Makoto Nishida, Hirotaka Kida, Hiroshi Handa, Hiroki Nishine, Masamichi Mineshita

**Affiliations:** ^1^ Division of Respiratory Diseases, Department of Internal Medicine St. Marianna University School of Medicine Kawasaki Japan

**Keywords:** ALK fusion gene‐positive lung cancer, brain metastasis, brigatinib, non‐small cell lung cancer (NSCLC), tyrosine kinase inhibitors (TKIs)

## Abstract

Anaplastic lymphoma kinase (ALK) fusion gene‐positive lung cancer often shows brain metastasis at initial diagnosis or during the course of treatment. However, molecular‐targeted drugs are known to pass through the blood–brain barrier and present positive effects for central nervous system lesions. There are few reports suggesting how effective molecular‐targeted drug therapy alone is for brain metastasis lesions of ALK fusion‐positive lung cancer, especially after the first use of ALK‐tyrosine kinase inhibitor (TKI) or for bulky brain metastases. A patient in his mid‐fifties with stage IV pleural dissemination developed brain metastases after 10 years of crizotinib use, but showed a complete response after switching to brigatinib. Moreover, a patient in her early sixties with stage III recurrent large brain metastases 5 years after chemoradiation therapy experienced dramatic tumor shrinkage with brigatinib. In each case of ALK fusion gene‐positive lung cancer with brain metastases, brigatinib showed a high efficacy and was well‐tolerated after previous ALK‐TKI and for bulky lesions.

## Graphical abstract

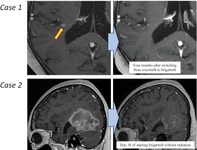

## INTRODUCTION

Molecular‐targeted drugs are known to pass through the blood–brain barrier (BBB) and present high effects on central nervous system (CNS) lesions.^1^ Non‐small cell lung cancer (NSCLC) often shows brain metastasis at the time of initial diagnosis or during the course of treatment, and this frequency is particularly high in patients with anaplastic lymphoma kinase (ALK) fusion gene‐positive lung cancer.^2^ The management of brain metastasis is important because it directly affects the patient's prognosis, and various treatment methods are indicated depending on the general condition of the patient and the number and size of brain metastasis. Treatment methods include surgery, local or whole brain irradiation, cytotoxic chemotherapy and molecular‐targeted drugs. Conversely, it has been clarified that there are multiple types of molecular‐targeted agents, and their therapeutic effects on brain metastasis differ.^2^, ^3^, ^4^ In this study, we introduce two important cases in which brain metastases were successfully treated with the use of brigatinib, which is the most recent ALK‐tyrosine kinase inhibitor (TKI) released in Japan.

## CASE SERIES

**FIGURE 1:**
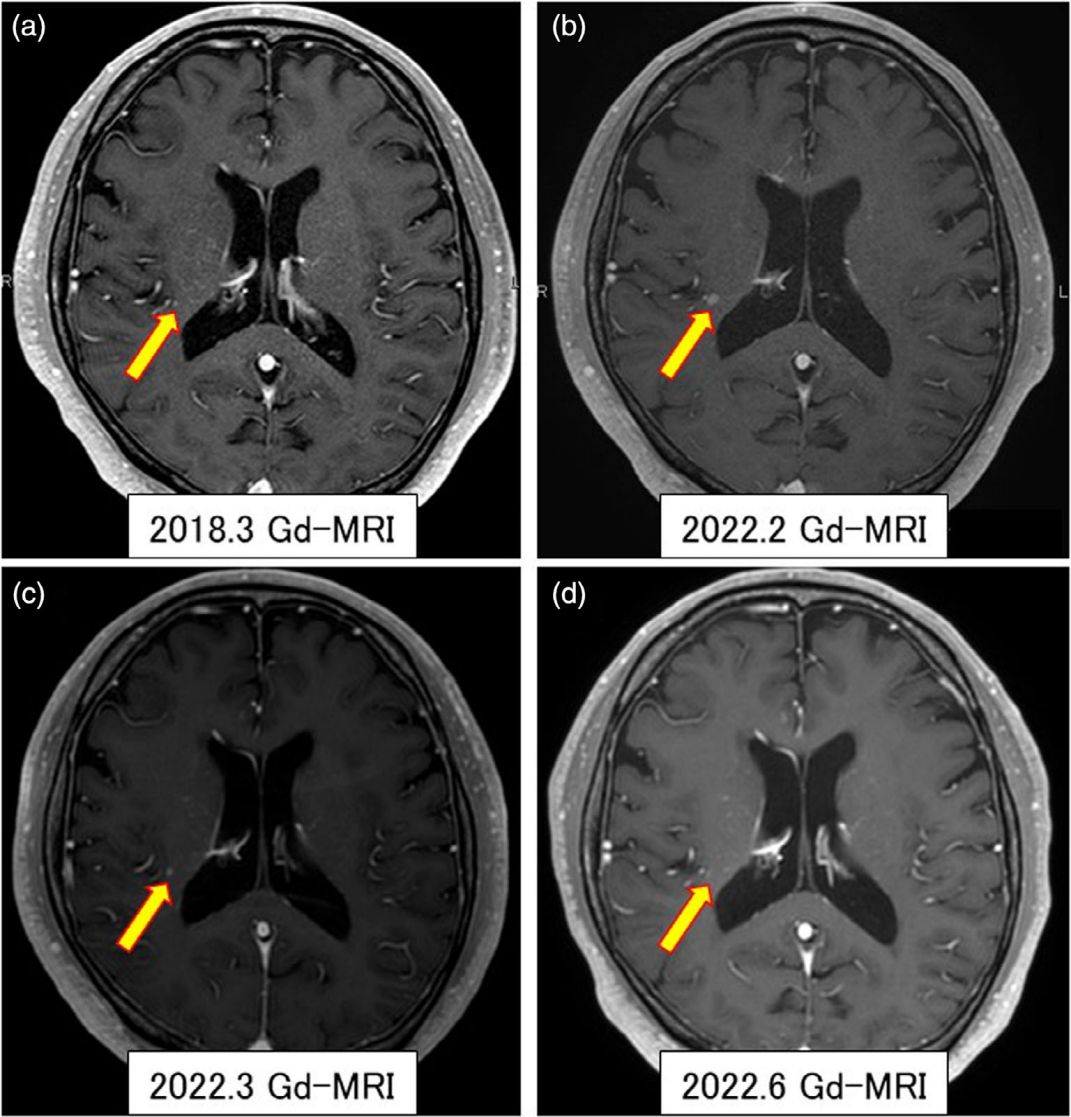
Clinical course of a single brain metastasis. Gradual progression during crizotinib administration (a, b), a reduction, then a disappearance of the lesion after switching to brigatinib (c, d). The yellow arrows point to the lesion.

The initial case was a male, never smoker, in his mid‐fifties with a history of hyperlipidemia and hypertension. He was referred to our department as an abnormality had been detected on chest computed tomography (CT) and was subsequently diagnosed with clinical stage I right lower lobe adenocarcinoma. He subsequently underwent surgery in 2012. Pleural dissemination was confirmed during the operation and the primary lesion was resected. The resected tumor was confirmed positive for EML4‐ALK fusion gene, and he was treated with crizotinib for about 10 years. However, a new asymptomatic single brain metastasis emerged in 2022 (Figure 1b), and a retrospective search discovered a very small nodule four years earlier (Figure 1a). By changing the molecular‐targeted drug from crizotinib to brigatinib, the brain metastasis was reduced (Figure 1c) and a complete response was achieved (Figure 1d). The oral administration of brigatinib has continued without any adverse events requiring a dose reduction or discontinuation.

**FIGURE 2:**
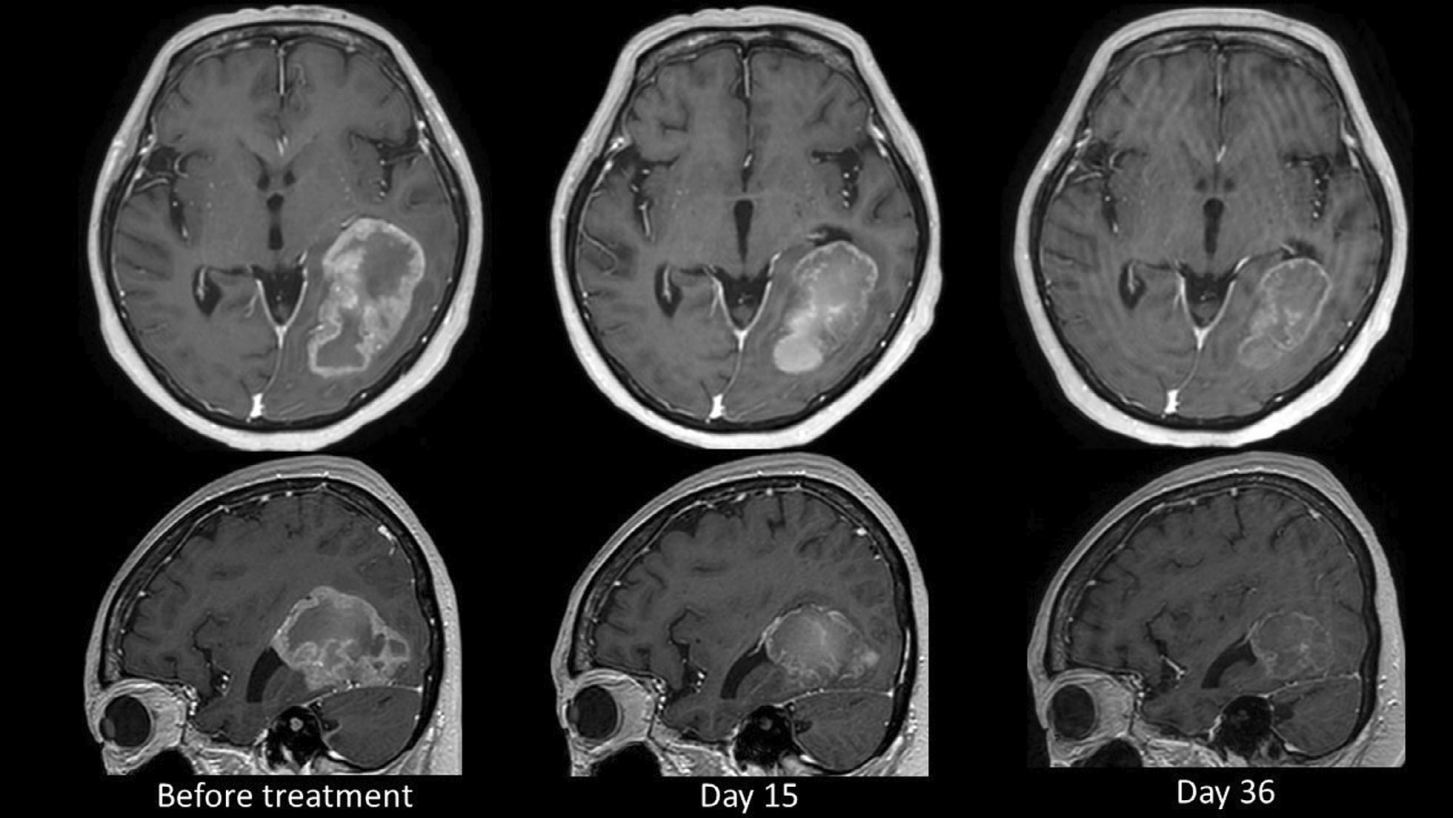
A bulky brain metastasis shrank rapidly and markedly after brigatinib administration.

The second patient was female and in her early sixties who had completed chemoradiotherapy for stage III ALK‐positive lung adenocarcinoma. She had been absent for regular outpatient visits but presented to the emergency department with dysphagia and a worsening performance status. CT/magnetic resonance imaging (MRI) revealed a bulky brain metastasis with a length of 70 mm. Based on her clinical course, brain metastasis from ALK lung cancer was suspected. However, a direct biopsy from the brain metastasis was not performed due to a brain puncture being high risk. Paralysis of the right vocal cord due to recurrent laryngeal nerve palsy was also present. At the request of the patient and her family, radiation therapy was declined, and only molecular‐targeted drug administration was indicated. A brain‐enhanced MRI was performed after brigatinib administration of 2 and 5 weeks, which showed a dramatic reduction of the large metastatic brain tumor (Figure 2). Although the right vocal cord paralysis remained, the patient was transferred to outpatient treatment. In this case, hepatic enzyme elevation to grade 2 was observed, but this improved a few days after drug withdrawal. Brigatinib was continued without dose reduction.

## DISCUSSION

ALK fusion gene‐positive lung cancer often develops at a young age, and a strategy to prolong survival through sequential treatment based on molecular‐targeted drugs is important.^1^ ALK fusion gene‐positive lung cancer has a high rate of complication with brain metastasis, with 34% reported to have brain metastasis at the time of diagnosis.^5^ Furthermore, the number of brain metastases has been reported to increase during the course of treatment.^6^ It has also been reported that ALK fusion gene‐positive lung cancer causes symptoms and worsens quality of life (QOL) in patients with brain metastases.^7^, ^8^ However, some prospective clinical trials have reported the cumulative incidence of brain metastasis was suppressed by the use of molecular‐targeted drugs.^3^, ^4^, ^9^ Hence, the choice of molecular‐targeted drugs is important. Among ALK‐TKIs, a first‐line crizotinib‐versus‐alectinib trial yielded major advances in control of brain metastases in the alectinib arm.^3^ Brigatinib, a second‐generation ALK‐TKI, was developed with an emphasis on its ability to penetrate the central nervous system. It has also shown promising results for the hazard ratio (HR) in comparison studies with crizotinib.^4^ For intention‐to‐treat analysis, the HR for overall survival was 0.81 in the brigtinib and crizotinib groups, whereas in subgroup analysis, the HR in the presence of brain metastases at initial presentation was 0.43 in the brigtinib and crizotinib groups.^9^ It has been suggested that the use of these drugs are highly effective against brain lesions and can improve prognosis if brain metastasis is present at the first visit.

There are multiple factors that contribute to the penetration of drugs through the BBB. First, the difference of blood concentration of drug, that is, the difference of the administration dose compared with the maximum tolerance dose. This has been well‐discussed and compared for gefitinib, erlotinib and osimertinib in EGFR‐TKIs.^10^, ^11^ Other factors include the affinity of the drug for BBB and the difference in the organic nature of the drug from that of P‐glycoprotein.^12^

In the cases reported here, we were able to confirm the positive effect of brigatinib on brain metastases. The first case was found to have pleural dissemination and was perscribed crizotinib over a long period of time. It was suspected that brain metastasis developed during the administration of crizotinib. It is unclear whether the brain metastasis occurred because of the poor drug penetration of crizotinib or if a resistance to crizotinib occured. However, the change of drug to brigatinib resulted in a complete response for the brain lesions. Thus, this is an important case series to demonstrate control of brain lesions by altering molecular‐targeted drugs. It has been reported that changing to ALK‐TKI may control brain lesions with a 25% response rate and 88% disease control rate.^13^ In this case series, a complete response was obtained by changing drug therapy. On the other hand, stereotactic radiotherapy is another standard treatment for small and few brain metastases. However, there is no clear evidence as to which therapy should be given first.^14^, ^15^ In our first case, the patient had been taking crizotinib continuously for nearly 10 years, and there was concern about the acquisition of resistance. Therefore, we first changed treatment to brigatinib. Since complete remission was achieved for a single brain metastasis, follow‐up is currently underway without additional irradiation.

Our second case was valuable as it included a large metastatic brain tumor of 70 mm or more which was sufficiently controlled by only molecular‐targeted drugs. In addition, in situations where oral administration is difficult, administering drugs through a gastric tube should be considered.^16^ Even when the general condition of the patient is poor, treatment should be considered after sufficiently explaining the risks of adverse events.

Due to the rarity of ALK fusion gene‐positive lung cancer, the limitation of this study is that we have only reported the efficacy of brigatinib for brain metastases in only two cases. Currently, a prospective multicenter phase 2 study is ongoing in Japan regarding the efficacy of brigatinib for brain metastases.^17^

In the treatment of ALK fusion gene‐positive lung cancer, it is important to use molecular‐targeted drugs and cytotoxic anticancer drugs sequentially. Furthermore, a change in treatment based on acquired resistance mutations,^18^ and the early detection of disease progression are crucial issues in the future.^19^, ^20^

In conclusion, in two cases of ALK lung cancer with brain metastases, brigatinib showed a high efficacy and was well‐tolerated for bully lesions and after the use of other ALK‐TKI.

## AUTHOR CONTRIBUTIONS

K. M. had full access to data in this case report and takes responsibility for the integrity and accuracy of data analysis. Y.N., Y. S., S. K., A. M., M. N., H. K., H. H., H. N., and M. M. contributed to the scientific review and final approval of this manuscript. All authors read and approved the final manuscript.

## CONFLICT OF INTEREST STATEMENT

The authors have no conflicts of interest to declare.
